# Within-host evolution drives the emergence of ceftazidime-avibactam resistance mediated by IncN plasmid-encoded *bla*_NDM-1_ and *bla*_KPC-33_ in ST11-KL64 hypervirulent *Klebsiella pneumoniae*

**DOI:** 10.1128/spectrum.02367-25

**Published:** 2026-02-19

**Authors:** Peiwen Xia, Na Huang, Tingting Si, Shiyu Tang, Qi Han, Yuqiong Li, Yuanyuan Song, Yingyi Liu, Yun Xia

**Affiliations:** 1 Department of Laboratory Medicine, The First Affiliated Hospital of Chongqing Medical University 117972, Chongqing, China; Tel Aviv Sourasky Medical Center, Tel Aviv, Israel

**Keywords:** KPC-producing hypervirulent *Klebsiella pneumoniae*, ceftazidime-avibactam, *bla*_NDM-1_, IncN, *bla*_KPC-33_, adaptive evolution

## Abstract

Ceftazidime-avibactam (CZA) is considered one of the most effective antimicrobial agents for treating infections caused by *Klebsiella pneumoniae* carbapenemase-producing *Klebsiella pneumoniae*. However, rising CZA resistance poses a critical clinical challenge, primarily driven by *bla*_KPC_ mutations. From a single CZA-treated patient, we isolated two CZA-susceptible and three CZA-resistant *K. pneumoniae* strains. Whole-genome sequencing of CZA-resistant strains CYH4028 and CYH1535 revealed two key resistance pathways: (i) acquisition of a conjugative IncN plasmid harboring *bla*_NDM-1_ and (ii) mutation of the *bla*_KPC-2_ gene to *bla*_KPC-33_ with an Ω-loop D179Y substitution. The *bla*_NDM-1_ gene resided within a truncated Tn*125* transposon that formed part of an IS*26*-associated translocatable unit in the self-transferable IncN plasmid pCYH4028_NDM. Phylogenetic analysis of 257 IncN plasmids revealed the highest plasmid prevalence in China (34.6%), with *K. pneumoniae* as the predominant host (41.6%) and *bla*_KPC_ as the most frequent carbapenemase gene (36.2%). Conjugation assays further confirmed self-transferability of pCYH4028_NDM. The *bla*_KPC-33_ gene was located within an IS*26*-truncated Tn*6296* transposon on the hybrid plasmid pCYH1535_Vir_KPC, which formed via IS*5075*-mediated homologous recombination and XerD-driven site-specific recombination. Notably, acquisition of CZA resistance incurred varying *in vitro* fitness costs, whereas the *bla*_KPC-33_-harboring strain exhibited greater competitive advantage than the *bla*_NDM-1_-harboring strain. All strains exhibited attenuated virulence (reduced mucoviscosity and biofilm production) due to IS*Kpn26* disruption of *rmpA*, a mucoid phenotype regulator. These findings elucidate within-host adaptive evolution in ST11-KL64 hypervirulent carbapenem-resistant *K. pneumoniae* and highlight the necessity for continuous genomic surveillance during therapy.

**IMPORTANCE:**

The alarming rise of ceftazidime-avibactam (CZA) resistance, primarily through *bla*_KPC_ mutations, constitutes a critical threat to global health. Here, we reported the within-host evolution of CZA resistance, driven by IncN plasmid-encoded *bla*_NDM-1_ and *bla*_KPC_ mutations, in a high-risk ST11-KL64 hypervirulent carbapenem-resistant *Klebsiella pneumoniae* lineage during patient therapy. Our study demonstrates the emergence of simultaneous, independent evolutionary pathways under CZA pressure. Crucially, an IncN *bla*_NDM-1_ plasmid acted as a helper plasmid, facilitating co-transfer of *bla*_NDM-1_ with *bla*_KPC-2_ and virulence genes. These direct insights highlight the urgent need for enhanced genomic surveillance against this evolving threat.

## INTRODUCTION

Carbapenem-resistant *Klebsiella pneumoniae* (CRKP), exhibiting extensive drug resistance, has emerged as a critical challenge to infection treatment in healthcare settings (1, 2). This resistance is primarily mediated by plasmid-encoded carbapenemases, with *Klebsiella pneumoniae* carbapenemase-2 (KPC-2) being most prevalent and New Delhi metallo-β-lactamase (NDM) following (3, 4). In contrast, hypervirulent *K. pneumoniae* (hvKP) typically causes severe community-acquired infections but retains antibiotic susceptibility, relying on a conserved pLVPK-like virulence plasmid carrying markers, including *rmpA*, *rmpA2*, *iucA*, *iroB*, and *peg-344* (5). Of concern, the convergence of carbapenem resistance and hypervirulence has driven the rapid dissemination of hybrid strains (CR-hvKP or hypervirulent carbapenem-resistant *Klebsiella pneumoniae* [hv-CRKP]), predominantly through horizontal plasmid transfer (1, 6). Notably, a recent surveillance study in China has identified the ST11-KL64 subclone as the predominant hv-CRKP lineage, driving nosocomial outbreaks (7).

The rapid global dissemination of hv-CRKP has drastically limited effective treatment options, highlighting the urgent need for new antimicrobial agents. Ceftazidime-avibactam (CZA), a β-lactam–β-lactamase inhibitor combination approved in China since 2019, demonstrates excellent *in vitro* activity against carbapenemase-producing *Enterobacterales* and is clinically indicated for complicated urinary tract and intra-abdominal infections (8, 9). Notably, although ineffective against metallo-β-lactamase-producing strains, CZA serves as a first-line therapy for CRKP infections due to its potent inhibition of Ambler class A (e.g., KPC) and some class D (e.g., OXA-48) carbapenemases (10, 11). Nevertheless, widespread clinical use of CZA has coincided with increasing resistance, predominantly mediated by *bla*_KPC_ mutations. Novel *bla*_KPC_ variants derived from *bla*_KPC-2_ or *bla*_KPC-3_ are increasingly reported worldwide. Among these variants, *bla*_KPC-33_ has emerged as the predominant clinical variant in recent epidemiological surveillance. This derivative of *bla*_KPC-2_ carries an Ω-loop D179Y substitution that confers high-level resistance to CZA (12). A previous study demonstrated that multiple variants were identified within a single patient following CZA exposure. Although *bla*_KPC_ variants regained susceptibility to carbapenem antibiotics, treatment with these agents readily led to reversion to the *bla*_KPC-2_. This characteristic poses significant challenges for both therapeutic strategies and current laboratory detection methods (13).

In this study, sequential isolates from a single patient revealed two distinct resistance pathways: horizontal acquisition of a conjugative IncN plasmid harboring *bla*_NDM-1_ and mutation of the *bla*_KPC-2_ gene to *bla*_KPC-33_. Additionally, the development of CZA resistance is accompanied by changes in genomic and phenotypic profiles. Our findings demonstrate that antibiotic-driven selection pressure promotes adaptive evolution of both resistance and virulence in hv-CRKP, necessitating intensive genomic surveillance during therapy to prevent therapeutic failure.

## MATERIALS AND METHODS

### Bacterial strains and identification

Five *K. pneumoniae* strains were analyzed in this study. These strains were isolated from distinct clinical specimens collected from a single patient admitted to the Respiratory Intensive Care Unit (RICU) at The First Affiliated Hospital of Chongqing Medical University (Chongqing, China). All strains were identified as *K. pneumoniae* using matrix-assisted laser desorption ionization-time of flight mass spectrometry (bioMérieux, Marcy-l'Étoile, France). In this study, carbapenemase-encoding genes (*bla*_KPC_ and *bla*_NDM_) and virulence genes (*rmpA*, *rmpA2*, *iucA*, *iroB*, and *peg-344*) were amplified by polymerase chain reaction (PCR) and then verified by Sanger sequencing. Primer sequences are shown in Table S1.

### Antimicrobial susceptibility test

The antimicrobial susceptibility testing (AST) was performed using the broth microdilution method, according to the Clinical and Laboratory Standards Institute (CLSI) M100 2023 guidelines (CLSI M100-S33, 2023). The following antimicrobial agents were tested, including amikacin, aztreonam, aztreonam-avibactam, ceftriaxone, ceftazidime, cefepime, ciprofloxacin, ceftazidime-avibactam, levofloxacin, imipenem, meropenem, piperacillin-tazobactam, and tigecycline. Avibactam was tested at a fixed concentration of 4 mg/L in combination with aztreonam or ceftazidime. For quality control, *Escherichia coli* ATCC 25922 was included in each experiment. Minimum inhibitory concentration results were interpreted using the 2023 CLSI breakpoints (CLSI M100-S33, 2023), with tigecycline susceptibility evaluated according to U.S. Food and Drug Administration criteria (https://www.fda.gov/drugs/development-resources/tigecycline-injection-products).

### Molecular genotyping and capsular typing of *K. pneumoniae* strains

Multilocus sequence typing (MLST) was performed according to the Pasteur Institute scheme (https://bigsdb.pasteur.fr/), targeting seven housekeeping genes (*gapA*, *infB*, *mdh*, *pgi*, *phoE*, *rpoB*, and *tonB*) to determine their sequence types. Capsular genotyping for serotype-specific alleles was performed by PCR using previously described methods (14).

### Whole-genome sequencing and bioinformatics analysis

Genomic DNA from *K. pneumoniae* strains was extracted using the Wizard Genomic DNA Purification Kit (Promega, Wisconsin) according to the manufacturer’s protocol. Hybrid whole-genome sequencing (WGS) was performed using a combined approach of long-read sequencing via PacBio RS II SMRT technology and short-read sequencing on an Illumina HiSeq X Ten platform. Library preparation and sequencing were provided by Majorbio Biopharm Technology Co., Ltd. (Shanghai, China). Genome assembly was performed using Unicycler (15), followed by protein-coding sequence prediction with Glimmer (16).

Plasmid replicon types, MLST, and capsular types were determined using the PlasmidFinder (https://cge.food.dtu.dk/services/PlasmidFinder/) and the BIGSdb *Klebsiella* genome (https://bigsdb.pasteur.fr/) databases. Core single-nucleotide polymorphisms (SNPs) were identified using Snippy v4.4.3 (https://github.com/tseemann/snippy) with *K. pneumoniae* CYH1535 as reference. A maximum-likelihood tree was then built from the alignment using FastTree (http://meta.microbesonline.org/fasttree/) and finally visualized and annotated in the iTOL (https://itol.embl.de/). Antimicrobial resistance genes, virulence genes, and insertion sequences were identified by aligning the assembled genomes against the CARD (https://card.mcmaster.ca/analyze/rgi), VFDB (http://www.mgc.ac.cn/VFs/main.htm), and ISfinder (https://www-is.biotoul.fr/blast.php). Plasmid conjugation modules, including *oriT*, relaxase, type IV coupling protein (T4CP), and type IV secretion system (T4SS), were predicted using oriTfinder2 (https://bioinfo-mml.sjtu.edu.cn/oriTDB2/oriTfinder.php) (17). Comparative genomic analyses were visualized with BRIG v0.95 for circular genome maps and Easyfig v2.2.3 for linear comparisons.

To explore the evolution of the IncN plasmid pCYH4028_NDM, a phylogenetic tree was constructed based on the core-genome backbones of similar IncN plasmids, using pCYH4028_NDM as the reference. Similar plasmids were defined as IncN plasmids from the NCBI database with >70% query coverage and >99% identity to pCYH4028_NDM. The tree of 257 plasmids was generated with kSNP4 based on core SNPs and visualized using iTOL (https://itol.embl.de/).

### Conjugation assay

The conjugative transferability of plasmid pCYH4028_NDM from the donor strain *K. pneumoniae* CYH4028 to the recipient *E. coli* EC600 (rifampicin[RIF] resistant) was assessed using a conjugation assay. Briefly, donor and recipient strains were cultured separately in 5 mL Luria–Bertani (LB) broth at 37°C with shaking (220 rpm) until reaching the logarithmic phase (optical density at 600 nm [OD_600_] approximately 0.6). Cells were mixed at a donor-to-recipient ratio of 1:2 (300 μL:600 μL), washed twice with PBS, and then plated onto LB agar containing CZA (8 μg/mL) and RIF (400 μg/mL). Following overnight incubation at 37°C, transconjugants were selected. Their identity was confirmed by ERIC-PCR genotyping and PCR amplification of *bla*_NDM-1_, *bla*_KPC-2_, and *iucA*.

### Growth curve determination and *in vitro* competition assay

To assess the *in vitro* fitness costs under non-competitive conditions, the growth kinetics of *K. pneumoniae* strains CYH4017, CYH4028, and CYH1535 were analyzed. Overnight LB broth cultures of each strain were standardized to a 0.5 McFarland turbidity unit (1 × 10^8^ CFU/mL), then diluted 100-fold in antibiotic-free LB broth and incubated at 37°C with shaking (220 rpm). Bacterial growth was monitored by measuring OD_600_ at 1-h intervals over a 12-h period.

To evaluate the relative fitness between strains, pairwise *in vitro* competition assays were conducted for *K. pneumoniae* CYH4017 vs CYH4028, CYH4017 vs CYH1535, and CYH4028 vs CYH1535. Overnight cultures in LB broth were normalized to a 0.5 McFarland standard and mixed at a 1:1 initial ratio in 5 mL LB broth. The co-cultures were incubated at 37°C with shaking (220 rpm) for 0, 24, and 48 h. Aliquots of serially diluted samples were plated on LB agar with or without meropenem (CYH4028 vs CYH1535) or ceftazidime/avibactam (CYH4017 vs CYH4028, CYH4017 vs CYH1535) at 0, 24, and 48 h to quantify viable cells. Strain-specific colony counts were determined by differential antibiotic selection. Selection competition index (CI) values were calculated as previously described (18). The competition was plotted with time as abscissa and CI as ordinate. A CI of 1 indicates no fitness advantage for the resistant strain; a CI of >1 indicates increased fitness of the resistant strain relative to the susceptible strain, while a CI of <1 indicates decreased fitness.

### Mucoviscosity and biofilm quantification

The hypermucoviscous phenotype of *K. pneumoniae* strains was assessed using a low-speed centrifugation assay. Overnight cultures were standardized to 0.5 McFarland turbidity, and 50 μL aliquots were inoculated into 5 mL fresh LB medium. After a 24-h incubation at 37°C with shaking at 220 rpm, 1 mL of culture was centrifuged at 1,000 × *g* for 5 min at room temperature. Data are presented as the OD_600_ post-centrifugation divided by OD_600_ pre-centrifugation. *K. pneumoniae* strain NTUH-K2044 and ATCC 700603 were served as positive and negative controls, respectively.

Biofilm formation was quantified using a crystal violet (CV) staining assay. Briefly, overnight cultures of test strains, NTUH-K2044 (positive control), and ATCC 700603 (negative control) were adjusted to 0.5 McFarland turbidity (1 × 10^8^ CFU/mL) and diluted 1:100 in fresh LB broth. Aliquots (200 μL) of each diluted suspension were transferred into 96-well polystyrene microplates and incubated at 37°C for 24 h. After incubation, planktonic cells were gently removed by aspiration, and adherent biofilms were washed three times with PBS. Biofilms were fixed by air-drying for 30 min, stained with 200 μL of 0.5% CV solution for 20 min, and washed three times with PBS to remove unbound dye. Bound CV was solubilized in 200 μL of 33% acetic acid for 30 min, and biofilm formation was quantified by measuring absorbance at OD_590_.

### Quantitative siderophore production assay

The relative siderophore production of strains was quantified using the chrome azurol S (CAS) assay in iron-depleted M9 minimal medium supplemented with casamino acids (c-M9-CA), as described previously. Overnight cultures were standardized to 0.5 McFarland turbidity (1 × 10^8^ CFU/mL), and 50 μL aliquots were inoculated into 5 mL fresh c-M9-CA medium. After 24 h incubation at 37°C with shaking (200 rpm), cultures were centrifuged at 10,000 × *g* for 10 min. Supernatants were passed through 0.22 μm filters, and 100 μL filtrates were mixed with 100 μL CAS shuttle solution in 96-well plates. Following a 60-min incubation in the dark at room temperature, absorbance was measured at OD_630_ nm (As), with uninoculated c-M9-CA medium as the reference blank (Ar). Percentage siderophore units were defined as [(Ar − As) / Ar] × 100%. Strains NTUH-K2044 and ATCC 700603 served as positive and negative controls, respectively. Three biological replicates were performed with triplicate technical measurements.

### Serum resistance assay

The serum resistance assay was performed as described previously with minor modifications (19). The mid-logarithmic phase strains were adjusted to 0.5 McFarland turbidity (1 × 10^8^ CFU/mL) and diluted to 1 × 10^6^ CFU/mL in LB broth. A 50 μL of each bacterial cell was mixed with 150 μL pooled normal human serum in 1.5 mL microcentrifuge tubes. The mixtures were incubated at 37°C with 220 rpm for 0, 60, 120, and 180 min. At each time point, aliquots were serially diluted and plated on LB agar. CFUs were enumerated after a 16–18 h incubation at 37°C. NTUH-K2044 and ATCC 700603 were used as positive and negative controls, respectively. Each strain was tested at least three times.

### *Galleria mellonella* infection models

The virulence of *K. pneumoniae* strains was assessed using a *Galleria mellonella* larvae model as previously described (20). *G. mellonella* larvae, weighing 200–300 mg, were randomly allocated into experimental groups containing 10 larvae each. Bacterial suspensions were prepared from mid-logarithmic-phase cultures and adjusted to 1 × 10⁶ CFU/mL in PBS. Subsequently, a 10 μL aliquot of each bacterial suspension was injected into the larvae, and inoculated larvae were maintained in the dark at 37°C. The survival rates were recorded at 12-h intervals over a 72-h observation period. Sterile PBS was used as the blank control; strain NTUH-K2044 and ATCC 700603 were used as hypervirulence and low-virulence controls, respectively. Each assay was independently repeated three times.

### Statistical analysis

Statistical analyses were performed using GraphPad Prism 10 software. One-way analysis of variance and log-rank (Mantel–Cox) tests were applied to determine statistical significance. ***P < 0.001, **P < 0.01, and *P < 0.05 are considered statistically significant.

## RESULTS

### Clinical information and antimicrobial resistance phenotype of hv-CRKP strains

**Fig 1:**
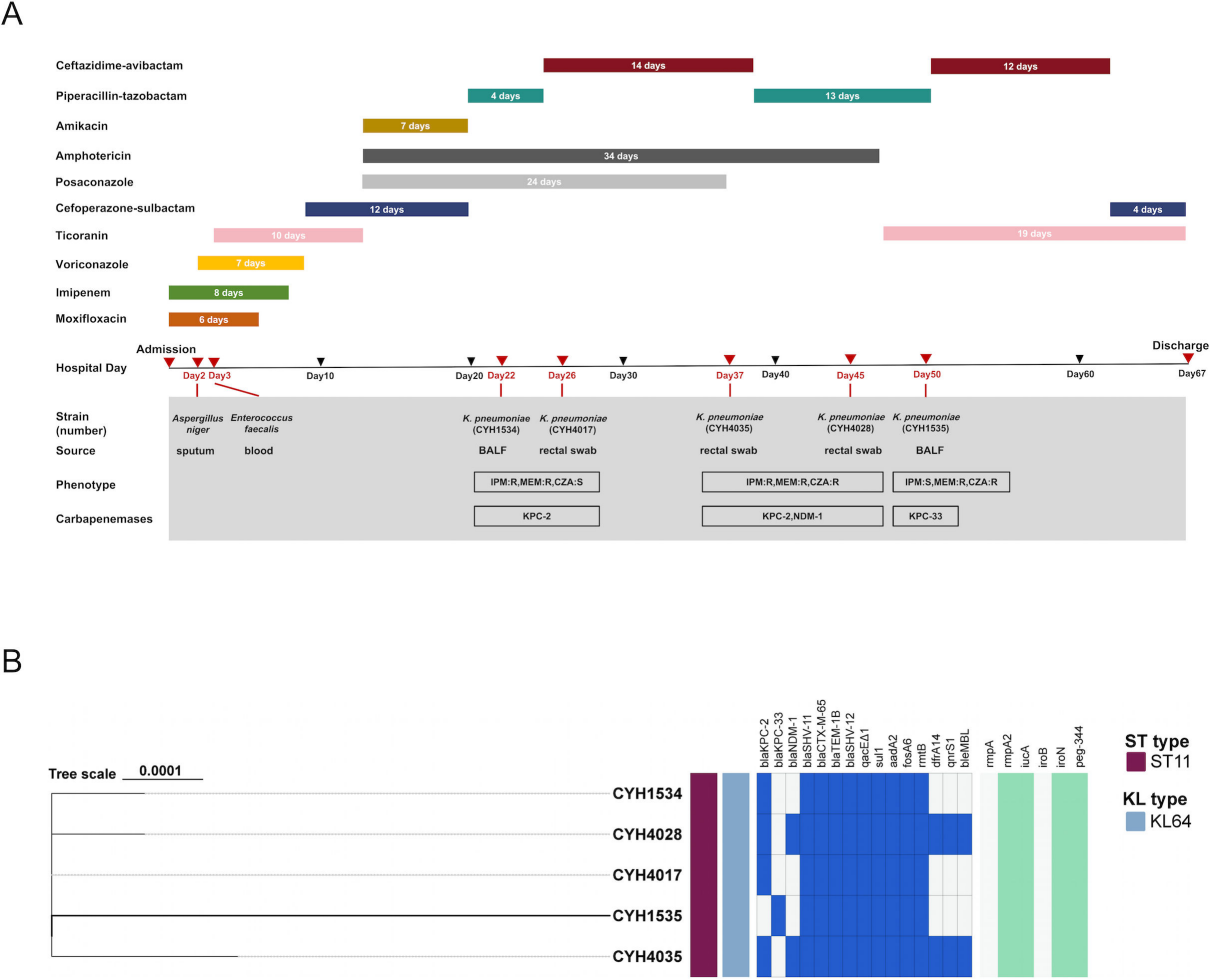
Timeline of infection during the hospital admission and clonal relatedness of clinical *Klebsiella pneumoniae* strains. (**A**) The timeline of antibiotic therapies and corresponding bacterial isolates during the clinical course of a single patient. BALF, bronchoalveolar lavage fluid; CZA, ceftazidime-avibactam; IPM, imipenem; MEM, meropenem; R, resistance; S, susceptibility. (**B**) Core-genome SNP phylogeny of the five *K. pneumoniae* isolates, annotated with their multilocus sequence types, capsular (KL) types, as well as resistance and virulence gene profiles.

A 61-year-old male with COVID-19-associated pneumonia was admitted to the RICU of The First Affiliated Hospital of Chongqing Medical University on 12 March 2024, after failed outside hospital treatment. He was diagnosed with severe pneumonia with respiratory failure. Figure 1A shows the treatment timeline and corresponding bacterial isolates.

Prior to isolating the CZA-susceptible *K. pneumoniae* CYH1534 from bronchoalveolar lavage fluid (BALF), the patient received combination therapy with cefoperazone-sulbactam (3.0 g q8h), amikacin (800 mg qd), and piperacillin-tazobactam (4.5 g q12h), during which inflammatory markers were elevated (PCT 6.62 ng/mL, leukocyte count 18.2 × 10⁹/L, and NEUT% 92.5%). Following AST results, the antimicrobial regimen was switched to CZA from day 25 to 38, which led to significant improvement in inflammatory parameters by day 38 (PCT 1.05 ng/mL, leukocytes 5.69 × 10⁹/L, and NEUT% 64.4%). Three CRKP strains were isolated from rectal swabs between days 26 and 45, with CYH4017 remaining CZA susceptible and CYH4035 and CYH4028 showing high-level CZA resistance. After CZA discontinuation, inflammatory markers rebounded by day 45 (PCT 1.53 ng/mL, leukocytes 13.87 × 10⁹/L, and NEUT% 82.2%). A CZA-resistant but carbapenem-susceptible *K. pneumoniae* (CYH1535) was isolated from BALF on day 50. Despite resumed CZA treatment, inflammatory parameters remained abnormal (PCT 2.75 ng/mL, leukocytes 13.87 × 10⁹/L, and NEUT% 81.6%). The patient was subsequently transferred to West China Hospital on 17 May 2024.

In addition to divergent resistance patterns to carbapenems (imipenem and meropenem) and CZA, AST revealed that all five *K. pneumoniae* strains were resistant to most β-lactam (including cephalosporins, aztreonam, and piperacillin-tazobactam), aminoglycosides, and fluoroquinolones, but retained susceptibility to tigecycline and aztreonam-avibactam (Table 1).

**TABLE 1:** Susceptibility of clinical *K. pneumoniae* strains, transformants, and recipients to antimicrobial agents

Antibiotics	MIC (µg/mL)								
	Donor					Recipient	Transconjugants		
	*K. pneumoniae* CYH1534	*K. pneumoniae* CYH4017	*K. pneumoniae* CYH4035	*K. pneumoniae* CYH4028	*K. pneumoniae* CYH1535	*E. coli* EC600	EC600-NDM (*bla*_NDM-1_)	EC600-NDM-KPC(*bla*_NDM-1,_ *bla*_KPC-2_)	ATCC 25922
Meropenem	64	64	>128	>128	4	≤0.03	16	32	≤0.03
Imipenem	64	16	128	128	2	0.125	16	32	0.125
Ceftazidime-avibactam	4/4	2/4	>256/4	>256/4	128/4	≤0.06/4	>256/4	>256/4	≤0.06/4
Ceftazidime	>256	>256	>256	>256	>256	0.5	>256	>256	0.25
Aztreonam-avibactam	1/4	1/4	1/4	2/4	2/4	0.125/4	0.125/4	0.25/4	≤0.03/4
Aztreonam	>128	>128	>128	>128	>128	0.25	0.25	>128	≤0.03
Ceftriaxone	>128	>128	>128	>128	>128	0.125	>128	>128	≤0.03
Cefepime	>128	>128	>128	>128	>128	≤0.03	64	64	≤0.03
Levofloxacin	64	64	128	128	64	0.125	2	2	≤0.03
Ciprofloxacin	>32	>32	>32	>32	>32	2	8	8	≤0.008
Amikacin	>512	>512	>512	>512	>512	2	4	2	4
Piperacillin-tazobactam	>512/4	>512/4	>512/4	>512/4	>512/4	2/4	128/4	>512/4	≤1/4
Tigecycline	2	2	2	1	1	0.125	0.125	0.125	0.125

### Molecular and genomic characterization of hv-CRKP strains

The analysis of MLST and capsular genotyping demonstrated that all strains belonged to the epidemic clone ST11 with KL64 capsular type. Phylogenetic analysis based on core-genome SNPs revealed a highly clonal population (Fig. 1B), with all strains differing by only six to eight SNPs from the reference strain CYH1535, thereby establishing a single genetic lineage despite divergent antimicrobial susceptibility profiles. These strains underwent changes in resistance genotypes against CZA and carbapenems during treatment. Resistance and virulence gene profiling demonstrated that five strains shared identical virulence markers but exhibited divergent resistance gene carriage. Specifically, strains CYH1534 and CYH4017 carried *bla*_KPC-2_; CYH4035 and CYH4028 co-harbored *bla*_KPC-2_ and *bla*_NDM-1_, while CYH1535 possessed the *bla*_KPC-33_ variant (derived from *bla*_KPC-2_).

WGS was performed on two representative CZA-resistant strains of CYH4028 and CYH1535. Table 2 summarizes their genomic structures and key molecular characteristics. Both strains exhibited a remarkably consistent profile of chromosomally encoded resistance and virulence genes. However, the two strains differed in plasmid structure and number. Strain CYH4028 harbored four plasmids: a virulence plasmid (pCYH4028_Vir), two carbapenemase-encoding plasmids carrying *bla*_KPC-2_ (pCYH4028_KPC) and *bla*_NDM-1_ (pCYH4028_NDM), respectively, and a ColRNAI plasmid (pCYH4028_ColRNAI). In contrast, strain CYH1535 contained a virulence and *bla*_KPC-33_ co-carrying plasmid (pCYH1535_Vir_KPC), a IncFII(pHN7A8)/IncR-type plasmid (pCYH1535_MDR), and a ColRNAI plasmid (pCYH1535_ColRNAI).

**TABLE 2:** Genomic characteristics of two ceftazidime-avibactam-resistant *K. pneumoniae* strains (CYH4028 and CYH1535)

	MLST serotype		Size (bp)	Replicon type	Resistance genes	Virulence genes	Conjugative modules
*K. pneumoniae* CYH4028	ST11-KL64						
		Chromosome	5,464,322	/^*a*^	*aadA2*, *fosA6*, *bla*_SHV-11_, *qacEΔ1*, *sul1*	*mrkABCDFHIJ*, *fimABCDEFGHIK*, *entABCDEFS*, *fepABCDG*, *iroEN*, *irp1*, *irp2*, *ybtAEPQSTUX*	/
		pCYH4028_Vir	175,989	IncHI1B(pNDM-MAR)/repB	/	*iucABCDiutA*, *rmpA2*, *iroN*, *peg-344*	*oriT* region
		pCYH4028_KPC	156,639	IncFII(pHN7A8)/IncR	*bla*_KPC-2_, *bla*_CTX-M-65_, *bla*_TEM-1B_, *bla*_SHV-12_, *rmtB*	/	/
		pCYH4028_NDM	59,486	IncN	bla_NDM-1_, dfrA14, qnrS1, ble_MBL_	/	*oriT* region, relaxase, T4CP, T4SS
		pCYH4028_ColRNAI	11,971	ColRNAI	/	/	Relaxase, T4CP
*K. pneumoniae* CYH1535	ST11-KL64						
		Chromosome	5,475,955	/	*aadA2*, *fosA6*, *bla*_SHV-11_, *qacEΔ1*, *sul1*	*mrkABCDFHIJ*, *fimABCDEFGHIK*, *entABCDEFS*, *fepABCDG*, *iroEN*, *irp1*, *irp2*, *ybtAEPQSTUX*	/
		pCYH1535_Vir-KPC	243,768	IncHI1B(pNDM-MAR)/repB	*bla*_KPC-33_	*iucABCDiutA*, *rmpA2*, *iroN*, *peg-344*	*oriT* region
		pCYH1535_MDR	67,184	IncFII(pHN7A8)/IncR	*bla*_CTX-M-65_, *bla*_TEM-1B_, *bla*_SHV-12_, *rmtB*	/	/
		pCYH1535_ColRNAI	22,439	ColRNAI	/	/	Relaxase, T4CP

^*a*^ "/” indicates not applicable.

### Genetic features, phylogenetic analysis, and transferability of pCYH4028_NDM

**Fig 2:**
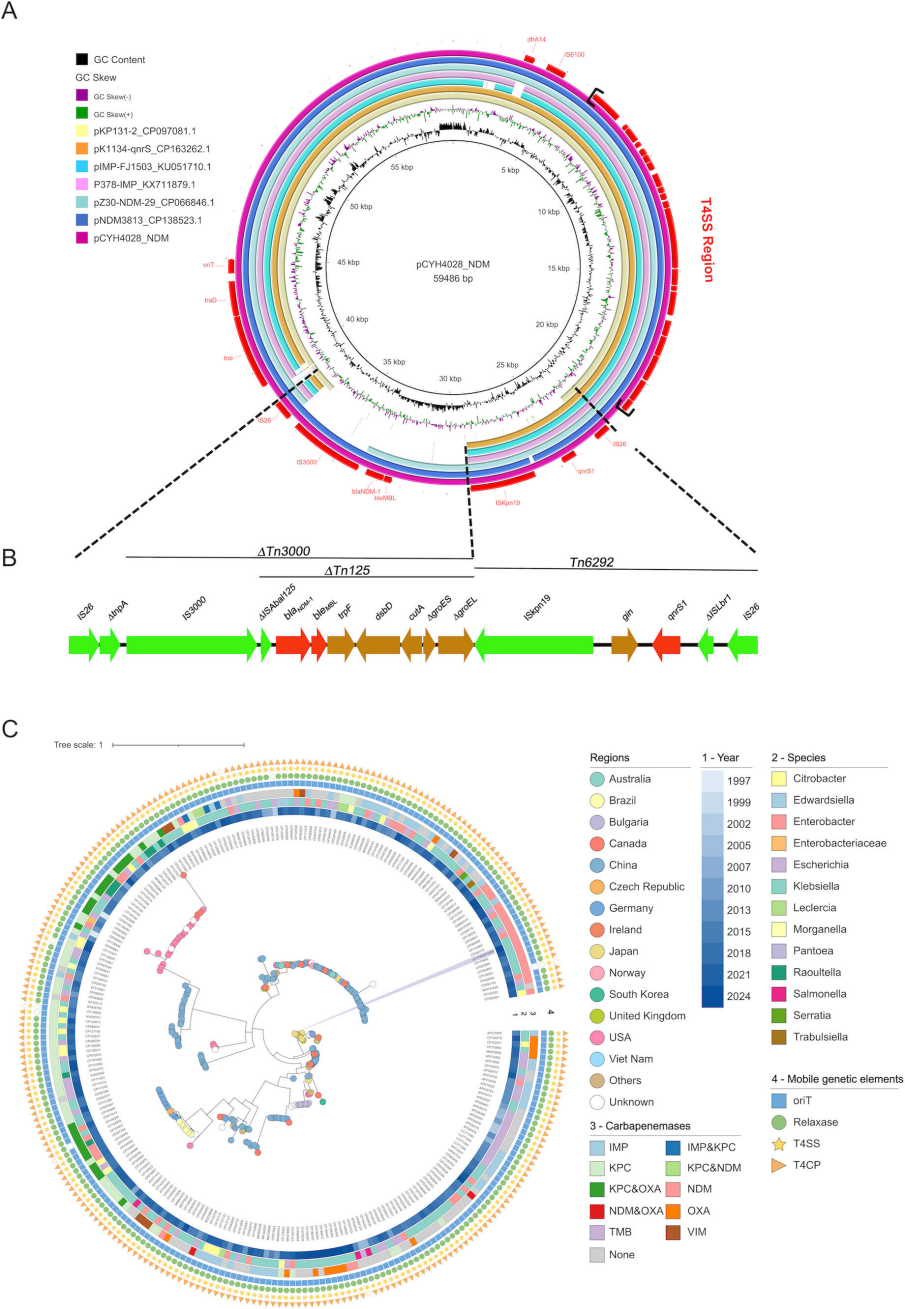
Alignment and phylogenetic analysis of pCYH4028_NDM. (**A**) Comparative analysis between the reference plasmid pCYH4028_KPC and other similar plasmids: рКР131-2_СР097081.1, pK1134-qnrS_CP163262.1, pIMP-FJ1503_KU051710.1, P378-IMP_KX711879.1, pZ30-NDM-29_CP066846.1, and pNDM3813_CP138523.1. The resistance genes and mobile genetic elements are indicated in red. (**B**) The genetic environment of *bla*_NDM-1_ in pCYH4028_NDM. The resistance genes are shown in red; mobile genetic elements are shown in green; open reading frames with specific functions are shown in yellow. (**C**) The phylogenetic tree of 257 IncN plasmids was constructed using pCYH4028_NDM as reference, with >70% query coverage and >99% identity. Plasmid details about regions, collection year, bacterial species, carbapenemases, and mobile genetic elements are shown.

**Fig 3:**
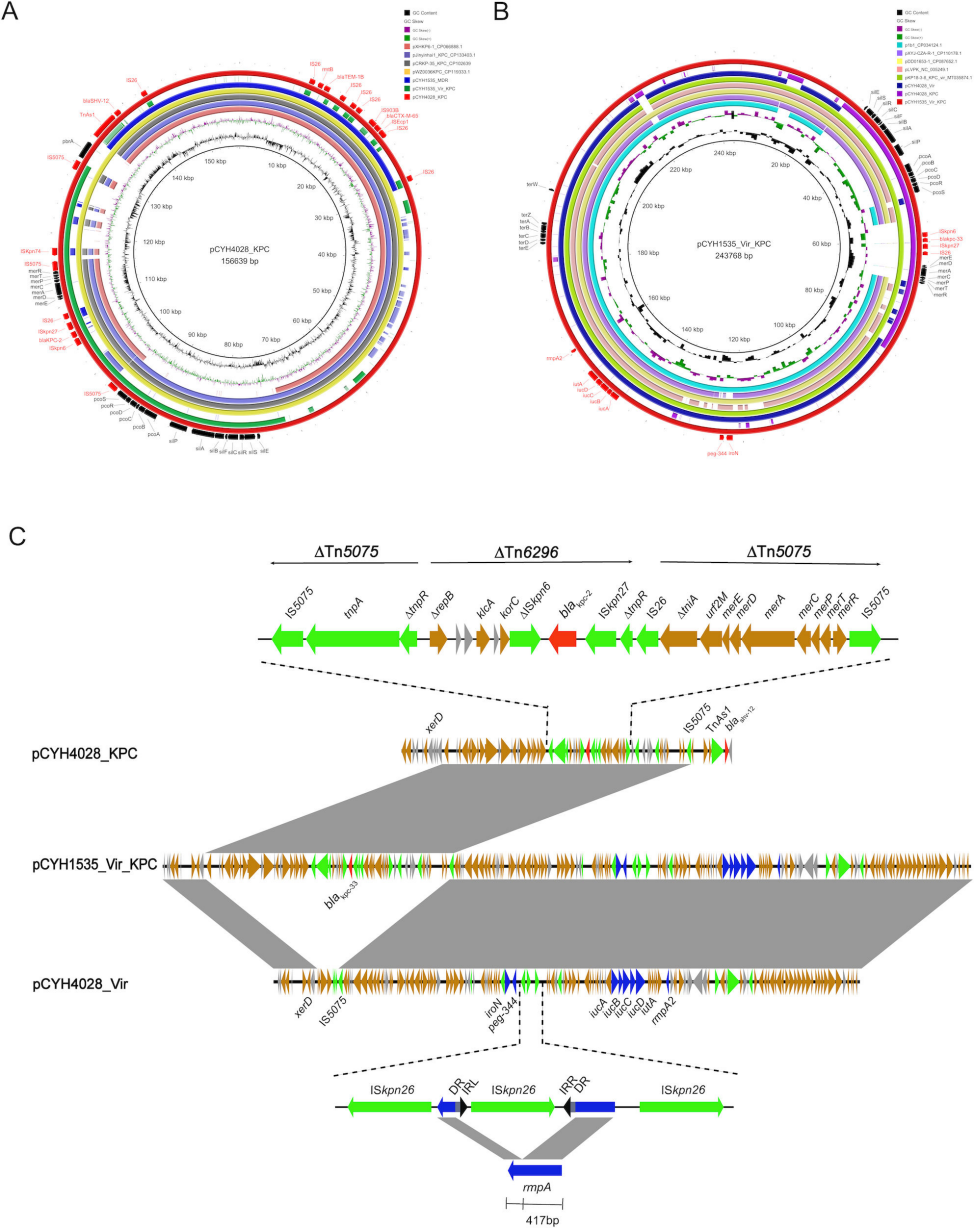
Comparative analysis of pCYH4028_KPC and pCYH1535_Vir_KPC with other similar plasmids. (**A**) Comparative analysis between the reference plasmid pCYH4028_KPC and other similar plasmids: pXHKP6-1_CPD66888.1, pJinyinhai1_KPC_CP133403.1, pCRKP-35_KPC_CP102639, pWZ0036KPC_CP119333.1, pCYH1535_MDR, and pCYH1535_Vir_KPC. The resistance genes and mobile genetic elements are indicated in red. (**B**) Comparative analysis between the reference plasmid pCYH1535_Vir_KPC and other similar plasmids: p1b1_СР034124.1, pXYJ-CZA-R-1_CP110178.1, pDD01653-1_CP087652.1, pLVPK_NC_005249.1, pKP18-3-8_KPC_vir_MT035874.1, pCYH4028_Vir, and pCYH4028_КРС. Virulence genes, resistance genes, and insertion sequences are indicated in red. (**C**) Linear comparisons of pCYH4028_KPC, pCYH1535_Vir_KPC, and pCYH4028_Vir. Genetic map of pCYH1535_Vir_KPC is composed of a 58-kb segment of pCYH4028_KPC and pCYH4028_Vir. The *bla*_KPC-2_ gene was located within a truncated Tn*6296* composite transposon. The *rmpA* gene was truncated by IS*Kpn26*. DR, direct repeat; IRL, left inverted repeat; IRR, right inverted repeat. The resistance genes are shown in red; virulence genes are shown in blue; mobile genetic elements are shown in green; open reading frames (ORFs) with specific functions are shown in yellow, and unidentified ORFs are shown in gray.

The *bla*_NDM-1_ gene was located on a 59,486 bp IncN plasmid, pCYH4028_NDM, with a GC content of 52.3%. BLASTN analysis showed >99% identity and 75%–100% query coverage to several previously reported plasmids (Fig. 2A). The plasmid carried a complete conjugation system, including *oriT*, relaxase, T4CP, and T4SS, indicating its self-transmissibility. The *bla*_NDM1_ genetic context consisted of an IS*3000* truncated Tn*125* transposon, forming the core structure ΔIS*Aba125–bla*_NDM-1_–*ble*_MBL_–*trpF–dsbD–cutA*–Δ*groES*–Δ*groEL*. This region was flanked by IS*26* elements and likely integrated into the plasmid backbone through IS*26*-mediated homologous recombination (Fig. 2B and 3A).

Phylogenetic analysis of pCYH4028_NDM revealed global dissemination of this plasmid backbone (Fig. 2C), with isolates reported in multiple countries, primarily in China (89, 34.6%) and the United States (34, 13.2%). These plasmids demonstrate a broad host range within *Enterobacterales*, predominantly found in *K. pneumoniae* (107, 41.6%) and *E. coli* (55, 21.4%). Notably, the IncN backbone harbors diverse carbapenemase genes, with *bla*_KPC_ (106, 36.2%), *bla*_IMP_ (51, 19.8%), and *bla*_NDM_ (28, 10.9%) being the most prevalent. Critically, 96.5% (248) of plasmids carry complete conjugative modules, highlighting the role of the IncN plasmid backbone in horizontal resistance gene dissemination.

Conjugation assays confirmed the transferability of plasmid pCYH4028_NDM. ERIC-PCR and targeted PCR verified the acquisition of two transconjugants, EC600-NDM and EC600-NDM-KPC (Fig. S1A and B). EC600-NDM carried *bla*_NDM-1_, while EC600-NDM-KPC co-harbored *bla*_NDM-1_, *bla*_KPC-2_, and *iucA*. WGS of EC600-NDM-KPC identified two recombinant plasmids. The 195,723 bp pEC600_Vir comprised sequences from pCYH4028_Vir and a 26-kb segment from pCYH4028_KPC (Fig. S2A). Linear comparison revealed that pEC600_NDM_KPC was a hybrid plasmid formed by pCYH4028_NDM and an IS*5075*-mediated 33-kb segment from pCYH4028_KPC (Fig. S2B). Furthermore, the 33-kb segment carrying *bla*_KPC-2_ underwent rearrangement during its integration into pCYH4028_NDM. Antibiotic susceptibility testing showed that EC600-NDM-KPC and EC600-NDM shared nearly identical resistance profiles, differing only in their susceptibility to aztreonam (Table 1).

### Generation of virulence and *bla*_KPC-33_ co-carrying plasmid pCYH1535_Vir_KPC

The *bla*_KPC-2_ gene was carried on pCYH4028_KPC, a 156,639 bp non-conjugative plasmid belonging to the IncFII (pHN7A8)/IncR incompatibility groups, which showed high similarity (90%–100% query coverage, >99% identity) to multiple known plasmids (Fig. 3A). In contrast, the *bla*_KPC-33_ gene was located on pCYH1535_Vir_KPC, a 243,768 bp IncHI1B(pNDM-MAR)/repB plasmid that also carried hypervirulence markers *rmpA2*, *iucABCD-iutA*, and *peg-344*. The plasmid shared high structural similarity with the classic virulence plasmid pLVPK (GenBank accession no. NC_005249.1). Unlike most similar plasmids, pCYH1535_Vir_KPC contained a 19-kb *bla*_KPC_-carrying segment. In addition, the plasmid contained an *oriT* region that enabled mobilization (Fig. 3B).

BLASTN comparison revealed pCYH1535_Vir_KPC to be a hybrid plasmid containing sequences from both pCYH4028_Vir and pCYH4028_KPC (Fig. 3B). Linear alignment further suggested that this plasmid likely originated from integration of a 58-kb segment of pCYH4028_KPC into the plasmid pCYH4028_Vir (Fig. 3C). Notably, all three plasmids carried *xerD*, a tyrosine recombinase gene associated with site-specific recombination. Additionally, the insertion sequence IS*5075* from pCYH4028_KPC was present in both pCYH4028_Vir and pCYH1535_Vir_KPC. Structural and sequence analyses indicate that plasmid fusion involved both homologous and site-specific recombination. Within the integrated region, *bla*_KPC-2_ was located in a Tn*6296*-related composite transposon truncated by IS*26*. The core genetic platform consisted of Δ*repB-hp-hp-klcA-hp-korC*-ΔIS*Kpn6-bla*_KPC-2_-IS*Kpn27*-Δ*tnpR*. Subsequently, the ΔTn*6296* transposon was inserted into a Tn*5075* transposon. Intriguingly, virulence gene analysis of pCYH4028_Vir indicated that *rmpA* was interrupted by the IS*Kpn26* sequence at position +417 bp, resulting in *rmpA* inactivation (Fig. 3C).

### The fitness cost and attenuated virulence of KPC-producing hvKP strains

**Fig 4:**
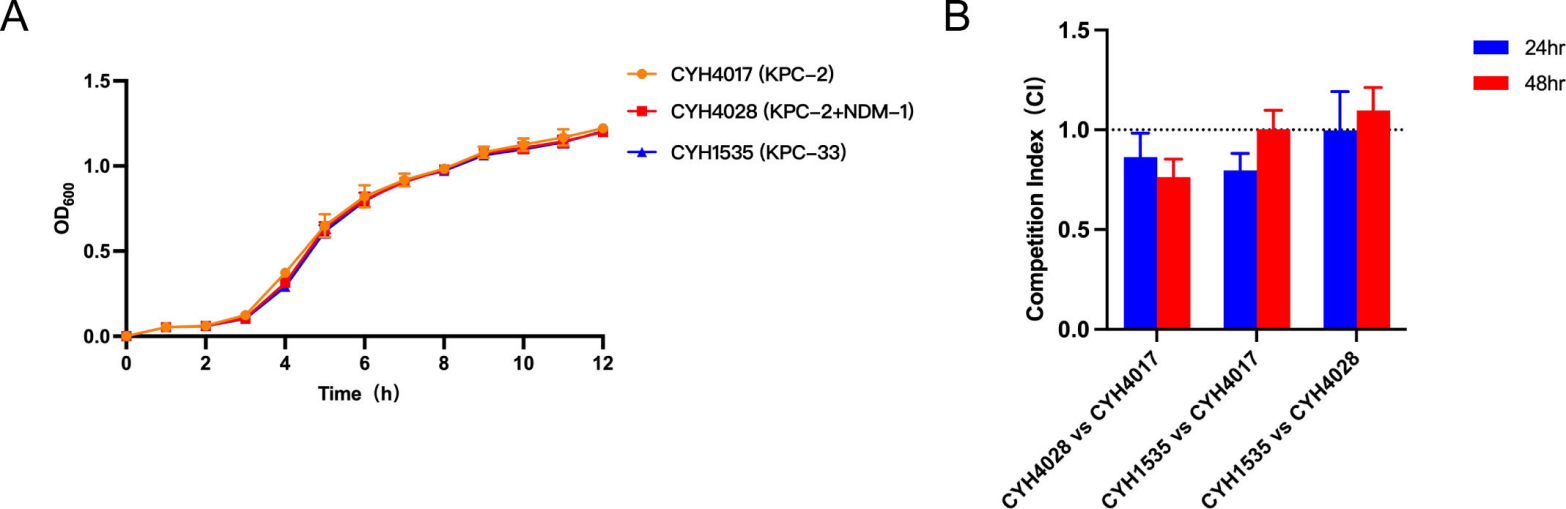
(**A**) The bacterial growth curve and (**B**) *in vitro* competition fitness between CZA-susceptible (CYH4017) and CZA-resistant strains (CYH4028 and CYH1535). The specific pairs are *K. pneumoniae* CYH4017 vs CYH4028, CYH4017 vs CYH1535, and CYH4028 vs CYH1535.

We then selected three strains, CYH4017, CYH4028, and CYH1535, to compare growth curves and *in vitro* competitive fitness between CZA-susceptible and CZA-resistant strains. Growth curves revealed that the three strains showed no significant difference in growth rates, suggesting that the acquisition of the *bla*_NDM-1_-bearing plasmid or *bla*_KPC-2_ mutation (conferring resistance to CZA) did not impose fitness costs on the strains in LB broth under non-competitive conditions (Fig. 4A). In contrast, competitive fitness assays produced divergent results. As shown in Fig. 4B, the CZA-resistant strain CYH4028 exhibited reduced fitness compared to the susceptible CYH4017 at both 24 and 48 h (CI < 1). Similarly, CYH1535 showed lower fitness than CYH4017 at 24 h (CI < 1), but its fitness was markedly increased at 48 h (CI = 1). Notably, direct competition between the two resistant strains (CYH1535 vs CYH4028) revealed parity at 24 h (CI = 1); however, CYH1535 showed a slightly increased fitness at 48 h (CI > 1).

**Fig 5:**
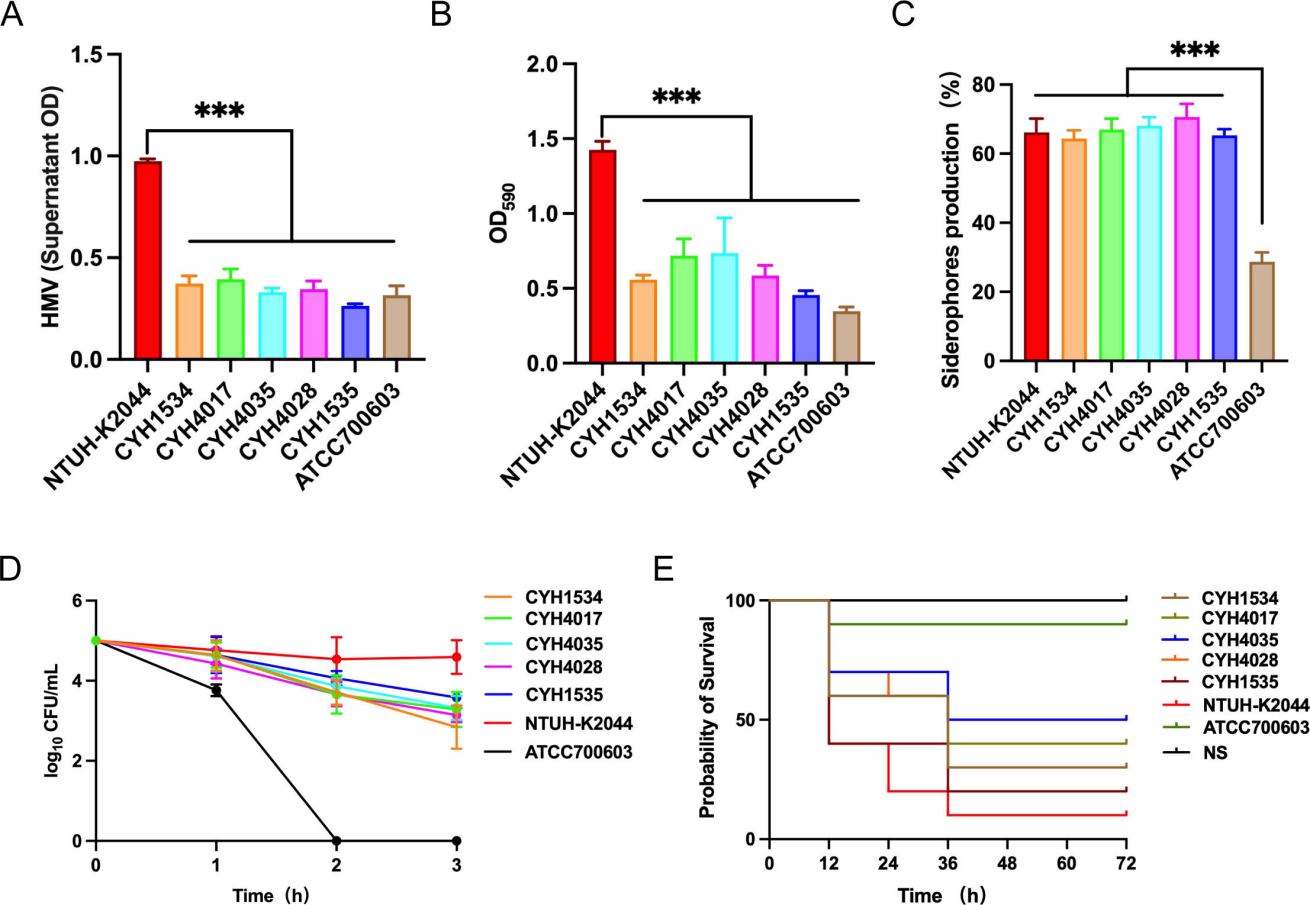
The virulence phenotypes and levels of five clinical *K. pneumoniae* strains. (**A**) Mucoid phenotype determined by mucoviscosity sedimentation assay. (**B**) Biofilm formation was quantified using a crystal violet staining assay. (**C**) Siderophore production measured by iron-chelated M9 minimal medium plus casamino acids. (**D**) Serum resistance assay. (**E**) The survival rates of strains in the *G. mellonella* infection model. NTUH-K2044 and ATCC 700603 were used as hypervirulence and low-virulence control, respectively. A one-way analysis of variance test was performed for mucoviscosity, biofilm formation, and siderophore production. A log-rank (Mantel–Cox) test was performed for the survival curves. ***P < 0.001, **P < 0.01, *P < 0.05.

To comprehensively assess the virulence potential of five *K. pneumoniae* clinical strains, we conducted comparative analyses against the low-virulence control ATCC 700603 and the hypervirulent control strain NTUH-K2044. The mucoviscosity sedimentation assay showed that all strains had significantly lower mucoviscosity than the NTUH-K2044, with levels comparable to those of ATCC 700603 (Fig. 5A). Similarly, biofilm formation assays demonstrated markedly reduced biofilm production in clinical strains relative to NTUH-K2044, but levels were comparable to ATCC 700603 (Fig. 5B). However, the clinical strains produced siderophore levels comparable to NTUH-K2044, significantly higher than ATCC 700603 (Fig. 5C). Notably, no significant differences in the assessed virulence phenotypes were observed among the five clinical strains. In the presence of pooled human serum, all strains were more susceptible to serum-mediated killing than NTUH-K2044 after the 180-min incubation (Fig. 5D). Furthermore, the virulence levels were verified using the *G. mellonella* larvae infection model (Fig. 5E). At 72 h post-infection, the five clinical strains showed host survival rates of 20%–50%, significantly lower than the 90% survival observed with ATCC 700603. In contrast, infection with NTUH-K2044 resulted in only 10% larval survival, indicating that the clinical strains exhibited attenuated virulence compared to the hypervirulent control.

## DISCUSSION

With the spread of carbapenem-resistant and hypervirulent *K. pneumoniae*, nosocomial infections have contributed to a growing burden in public health over the past few years (1, 21). The novel β-lactamase inhibitor AVI targets Ambler class A (e.g., KPC) and some class D (e.g., OXA-48) carbapenemases (12). Consequently, AVI is clinically used in combination with ceftazidime to treat complicated infections caused by KPC-producing *K. pneumoniae* (11). Since its clinical approval in China, CZA has become one of the most effective antimicrobial agents for such infections, significantly reducing mortality rates in treated patients (22, 23). However, increased clinical use of CZA has led to the emergence of resistance. In a clinical surveillance study, we isolated two CZA-susceptible and three CZA-resistant *K. pneumoniae* strains from a single patient treated with CZA, demonstrating *in vivo* resistance development under drug-selective pressure.

Both KPC and NDM carbapenemases are among the most prevalent types in China, with high prevalence of co-occurrence in *Enterobacterales* (7, 24). The emergence of these dual carbapenemase producers has led to resistance against nearly all clinically used antimicrobials, including CZA. In this study, we analyzed CYH4035 and CYH4028, two clinical CZA-resistant *K. pneumoniae* strains. These strains were originally derived from KPC-2-producing *K. pneumoniae* and subsequently acquired a *bla*_NDM-1_-encoding plasmid. The *bla*_NDM_ genes are predominantly located on IncX3 plasmids, which are key vectors for their dissemination in hospital settings (25, 26). However, we identified a *bla*_NDM-1_-bearing plasmid pCYH4028_NDM that harbored an IncN replicon. Plasmid pCYH4028_NDM was a self-transferable plasmid that contained essential conjugative modules (27).

Conjugation experiments confirmed its self-transferability to *E. coli* EC600. In addition, we observed the co-transfer of *bla*_NDM-1_, *bla*_KPC-2_, and the virulence gene *iucA*. Linear comparison of the related plasmids delineates two potential trajectories for *bla*_KPC-2_ mobilization. Both originate from the integration of a 58-kb segment of pCYH4028_KPC into pCYH4028_Vir, forming the hybrid plasmid pCYH4028_Vir_KPC. The models diverge in the order of subsequent events. The first model proposes the initial transfer of pCYH4028_Vir_KPC alone to EC600 with the assistance of pCYH4028_NDM, followed by integration of the 33-kb *bla*_KPC-2_-carrying segment into pCYH4028_NDM. The second model proposes that this integration occurs prior to transfer, resulting in the co-transfer of both recombinant plasmids. The central distinction thus rests on the relative timing of recombination and conjugation.

Phylogenetic analysis revealed that IncN plasmid pCYH4028_NDM is self-transmissible across *Enterobacterales* species. More importantly, plasmid backbones of this type frequently carry diverse carbapenemase genes, underlining their role as critical resistance gene vectors. These findings highlighted the urgent need for surveillance of self-transmissible IncN plasmids in clinical settings.

Previous studies reported that mutations in the *bla*_KPC_ gene represent the primary mechanism conferring high-level resistance to CZA (10, 12). Notably, the number of reported *bla*_KPC_ variants has increased dramatically in recent years, with over 240 variants identified globally (https://www.ncbi.nlm.nih.gov/pathogens/refgene#blaKPC). The development of *bla*_KPC_ mutations is driven by the selective pressure from CZA therapy. This clinical use creates an environment that enriches for bacterial variants harboring genetic alterations in *bla*_KPC_, such as nucleotide substitutions, insertions, and deletions. Specific mutations, particularly those in functional regions like the Ω-loop that reduce drug binding affinity, are thereby selected, leading to the emergence and clonal expansion of CZA-resistant strains. Mutations associated with CZA resistance predominantly localize to three structural regions: the Ω-loop region (R164–D179), the 237–243 loop, and the 266–275 loop (10). The CZA-resistant strain CYH1535 isolated in this study harbored *bla*_KPC-33_, the clinically prevalent variant of *bla*_KPC-2_, characterized by a D179Y substitution in the Ω-loop. This substitution is typically associated with significantly increased susceptibility to carbapenems, especially imipenem (28, 29). A possible explanation involves impaired hydrolysis of carbapenems due to altered enzyme activity caused by the D179Y substitution in KPC-2. Critically, the emergence of this mutant in our patient was preceded by a 14-day course of CZA therapy. This clinical history directly implicates CZA selective pressure as the primary driver of this evolutionary event.

As previous studies reported, the *bla*_KPC_ and *bla*_NDM_ have been conventionally carried on separate plasmids (30, 31). The *bla*_KPC-33_ gene was located on the IncHI1B (pNDM-MAR)/repB plasmid pCYH1535_Vir_KPC, a pLVPK-like virulence plasmid. Plasmid analysis indicated that the *bla*_KPC-33_-carrying region likely originated from a 58-kb segment of the IncFII(pHN7A8)/IncR-type plasmid pCYH4028_KPC. The conjugation assay also showed the integration of the *bla*_KPC-2_-bearing region into the virulence plasmid. As previously reported, plasmid-borne *xerD* plays a significant role in horizontal transfer of resistant genes in *Acinetobacter* species (32, 33). In this study, the hybrid plasmid may have formed through homologous recombination mediated by IS*5075* and site-specific recombination promoted by *xerD*. A similar recombination pattern was previously reported for the IncX3-X4 hybrid plasmid in *E. coli* strain CQ02-121 (34). The Tn*4401* transposon is the most common *bla*_KPC_-containing mobile element, mediating gene transfer via plasmid integration (35). However, *bla*_KPC-33_ in plasmid pCYH1535_Vir_KPC was located within an IS*26*-truncated Tn*6296* transposon, classified as a non-Tn*4401* element (NET_KPC_-Ib). Recent studies reported an increasing prevalence of *bla*_KPC_-containing Tn*6296* transposons in *K. pneumoniae* strains, highlighting the dissemination risk of Tn*6296*-mediated *bla*_KPC_ gene transfer (26, 36). Finally, the ΔTn*6296* identified in this study was generated by the insertion of the core genetic platform of *bla*_KPC-2_ into Tn*5075*, contrasting with the previously reported Tn*1722* (37).

In this study, we characterized CZA-susceptible *K. pneumoniae* strains harboring *bla*_KPC-2_ that developed resistance via distinct pathways within a single patient. A previous study reported similar CZA resistance mechanisms but involved acquisition of pX3_NDM-5 harboring *bla*_NDM-5_ (38). Furthermore, *in vitro* competition assay revealed that acquisition of CZA resistance imposed varying degrees of fitness costs on the strains, whereas the strain with *bla*_KPC-33_ exhibited greater competitive advantage than the strain with bla_NDM-1_. Generally, capsule polysaccharides and siderophores are key virulence determinants in hvKP (5). However, all strains here exhibited attenuated virulence, manifested by reduced mucoviscosity and biofilm production, likely due to IS*Kpn26*-mediated disruption of mucoid regulator *rmpA*. Notably, the sustained high production of the aerobactin siderophore, which is encoded by the *iucABCD-iutA* operon, was maintained despite the attenuation of mucoid phenotypes. This preserved iron acquisition capacity likely accounts for the intermediate virulence observed in the *G. mellonella* infection model. Consistent with previous observations of *rmpA* inactivation mediated by IS elements, these findings suggest that IS-driven *rmpA* disruption may represent an adaptive evolutionary strategy in hv-CRKP (39, 40).

The primary limitation of this study is that only one case was investigated, which precluded prevalence assessment. Additionally, the retrospective nature prevented tracing the origin of the *bla*_NDM-1_-bearing plasmid. Based on the self-transferability of the identified plasmid and the epidemiological evidence, we propose that the most probable origin of the *bla*_NDM-1_ gene was a conjugative plasmid acquired from other *Enterobacterales* within the host’s gut microbiome. This hypothesis is supported by the initial isolation of the *bla*_NDM-1_-negative strain (CYH4017) from a rectal swab, suggesting that the horizontal gene transfer likely occurred in this complex microbial environment.

In conclusion, our findings demonstrated the complex within-host evolution of CRKP to CZA-resistant strains, involving a *bla*_KPC-2_ to *bla*_KPC-33_ mutation and acquisition of a self-transferable *bla*_NDM-1_-harboring plasmid. Furthermore, CZA resistance emergence was accompanied by genetic recombination events and hv-CRKP adaptive evolution. Therefore, continuous surveillance of the microbial characteristics of the strains during treatment is essential.

## Data Availability

The complete genome sequences of the five *K. pneumoniae* strains and the transconjugant EC600-NDM-KPC have been deposited in the NCBI database under BioProject accession numbers PRJNA1365442, PRJNA1273678, PRJNA1273682, and PRJNA1371601, respectively.

## SUPPLEMENTAL MATERIAL

Figure S1 spectrum.02367-25-s0001.tif The confirmation of transconjugants EC600-NDM and EC600-NDM-KPC.

Figure S2 spectrum.02367-25-s0002.tif Structure and recombination analysis of pEC600_Vir and pEC600_NDM_KPC.

Table S1 spectrum.02367-25-s0003.docx Primers used in this study.

ASM does not own the copyrights to Supplemental Material that may be linked to, or accessed through, an article. The authors have granted ASM a non-exclusive, world-wide license to publish the Supplemental Material files. Please contact the corresponding author directly for reuse.

## References

[1] Dong N, Yang X, Chan EW-C, Zhang R, Chen S. 2022. Klebsiella species: taxonomy, hypervirulence and multidrug resistance. EBioMedicine 79:103998. doi:10.1016/j.ebiom.2022.103998 35405387 PMC9010751

[2] Shi Q, Ruan Z, Zhang P, Hu H, Han X, Wang Z, Lou T, Quan J, Lan W, Weng R, Zhao D, Du X, Yu Y, Jiang Y. 2024. Epidemiology of carbapenem-resistant Klebsiella pneumoniae in China and the evolving trends of predominant clone ST11: a multicentre, genome-based study. J Antimicrob Chemother 79:2292–2297. doi:10.1093/jac/dkae227 38997220

[3] Li J, Wu W, Wu H, Huang J, Li Z, Wang J, Zhou Z, Wu M, Wu X, Zhao Y, Ren J. 2025. Rapid emergence, transmission, and evolution of KPC and NDM coproducing carbapenem-resistant Klebsiella pneumoniae. Microbiol Res 293:128049. doi:10.1016/j.micres.2025.128049 39798298

[4] Liu C, Dong N, Chan EWC, Chen S, Zhang R. 2022. Molecular epidemiology of carbapenem-resistant Klebsiella pneumoniae in China, 2016-20. Lancet Infect Dis 22:167–168. doi:10.1016/S1473-3099(22)00009-3 35092791

[5] Russo TA, Olson R, Fang C-T, Stoesser N, Miller M, MacDonald U, Hutson A, Barker JH, La Hoz RM, Johnson JR. 2018. Identification of biomarkers for differentiation of hypervirulent Klebsiella pneumoniae from Classical K. pneumoniae. J Clin Microbiol 56:e00776-18. doi:10.1128/JCM.00776-18 29925642 PMC6113484

[6] Tian D, Liu X, Chen W, Zhou Y, Hu D, Wang W, Wu J, Mu Q, Jiang X. 2022. Prevalence of hypervirulent and carbapenem-resistant Klebsiella pneumoniae under divergent evolutionary patterns. Emerg Microbes Infect 11:1936–1949. doi:10.1080/22221751.2022.2103454 35844192 PMC9359173

[7] Wang Q, Wang R, Wang S, Zhang A, Duan Q, Sun S, Jin L, Wang X, Zhang Y, Wang C, Kang H, Zhang Z, Liao K, Guo Y, Jin L, Liu Z, Yang C, Wang H, China Carbapenem-Resistant Enterobacterales N. 2024. Expansion and transmission dynamics of high risk carbapenem-resistant Klebsiella pneumoniae subclones in China: an epidemiological, spatial, genomic analysis. Drug Resist Updat 74:101083. doi:10.1016/j.drup.2024.101083 38593500

[8] Shirley M. 2018. Ceftazidime-avibactam: a review in the treatment of serious gram-negative bacterial infections. Drugs (Abingdon Engl) 78:675–692. doi:10.1007/s40265-018-0902-x 29671219

[9] Tumbarello Mario, Trecarichi EM, Corona A, De Rosa FG, Bassetti M, Mussini C, Menichetti F, Viscoli C, Campoli C, Venditti M, et al.. 2019. Efficacy of ceftazidime-avibactam salvage therapy in patients with infections caused by Klebsiella pneumoniae carbapenemase-producing K. pneumoniae. Clin Infect Dis 68:355–364. doi:10.1093/cid/ciy492 29893802

[10] Hobson CA, Pierrat G, Tenaillon O, Bonacorsi S, Bercot B, Jaouen E, Jacquier H, Birgy A. 2022. Klebsiella pneumoniae carbapenemase variants resistant to ceftazidime-avibactam: an evolutionary overview. Antimicrob Agents Chemother 66:e0044722. doi:10.1128/aac.00447-22 35980232 PMC9487638

[11] Tumbarello M, Raffaelli F, Giannella M, Mantengoli E, Mularoni A, Venditti M, De Rosa FG, Sarmati L, Bassetti M, Brindicci G, et al.. 2021. Ceftazidime-avibactam use for Klebsiella pneumoniae carbapenemase-producing K. pneumoniae infections: a retrospective observational multicenter study. Clin Infect Dis 73:1664–1676. doi:10.1093/cid/ciab176 33618353

[12] Ding L, Shen S, Chen J, Tian Z, Shi Q, Han R, Guo Y, Hu F. 2023. Klebsiella pneumoniae carbapenemase variants: the new threat to global public health. Clin Microbiol Rev 36:e0000823. doi:10.1128/cmr.00008-23 37937997 PMC10732083

[13] Shi Q, Han R, Guo Y, Yang Y, Wu S, Ding L, Zhang R, Yin D, Hu F. 2022. Multiple novel ceftazidime-avibactam-resistant variants of bla(KPC-2)-positive Klebsiella pneumoniae in two patients. Microbiol Spectr 10:e0171421. doi:10.1128/spectrum.01714-21 35588280 PMC9241591

[14] Zhang Y, Zeng J, Liu W, Zhao F, Hu Z, Zhao C, Wang Q, Wang X, Chen H, Li H, Zhang F, Li S, Cao B, Wang H. 2015. Emergence of a hypervirulent carbapenem-resistant Klebsiella pneumoniae isolate from clinical infections in China. J Infect 71:553–560. doi:10.1016/j.jinf.2015.07.010 26304687

[15] Wick RR, Judd LM, Gorrie CL, Holt KE. 2017. Unicycler: resolving bacterial genome assemblies from short and long sequencing reads. PLoS Comput Biol 13:e1005595. doi:10.1371/journal.pcbi.1005595 28594827 PMC5481147

[16] Delcher AL, Bratke KA, Powers EC, Salzberg SL. 2007. Identifying bacterial genes and endosymbiont DNA with glimmer. Bioinformatics 23:673–679. doi:10.1093/bioinformatics/btm009 17237039 PMC2387122

[17] Liu G, Li X, Guan J, Tai C, Weng Y, Chen X, Ou HY. 2025. oriTDB: a database of the origin-of-transfer regions of bacterial mobile genetic elements. Nucleic Acids Res 53:D163–D168. doi:10.1093/nar/gkae869 39373502 PMC11701681

[18] Chou SH, Chuang C, Juan CH, Ho YC, Liu SY, Chen L, Lin YT. 2024. Mechanisms and fitness of ceftazidime/avibactam-resistant Klebsiella pneumoniae clinical strains in Taiwan. Int J Antimicrob Agents 64:107244. doi:10.1016/j.ijantimicag.2024.107244 38925227

[19] Tian D, Wang W, Li M, Chen W, Zhou Y, Huang Y, Sheng Z, Jiang X. 2021. Acquisition of the conjugative virulence plasmid from a CG23 hypervirulent Klebsiella pneumoniae strain enhances bacterial virulence. Front Cell Infect Microbiol 11:752011. doi:10.3389/fcimb.2021.752011 34604119 PMC8485780

[20] Liu H, Xiang Y, Xiong M, Xiao X, Zhou J, Tian H, Chen Q, Li Y. 2024. Prevalence of ST1049-KL5 carbapenem-resistant Klebsiella pneumoniae with a bla_KPC-2_ and bla_NDM-1_ co-carrying hypertransmissible IncM1 plasmid. Commun Biol 7:695. doi:10.1038/s42003-024-06398-w 38844513 PMC11156905

[21] Hu F, Pan Y, Li H, Han R, Liu X, Ma R, Wu Y, Lun H, Qin X, Li J, Wang A, Zhou M, Liu B, Zhou Z, He P. 2024. Carbapenem-resistant Klebsiella pneumoniae capsular types, antibiotic resistance and virulence factors in China: a longitudinal, multi-centre study. Nat Microbiol 9:814–829. doi:10.1038/s41564-024-01612-1 38424289 PMC10914598

[22] Chen TY-T, Hsu C-K, Shih S-C, Weng T-S, Tang H-J, Lai C-C. 2023. Comparing novel antibiotics and carbapenems for complicated intra-abdominal infections: a systematic review and meta-analysis of randomized controlled trials. Int J Antimicrob Agents 62:106844. doi:10.1016/j.ijantimicag.2023.106844 37160243

[23] Hung K-C, Tsai W-W, Hsu C-W, Lai C-C, Tang H-J, Chen I-W. 2023. Clinical efficacy and safety of novel antibiotics for complicated urinary tract infection: a systematic review and meta-analysis of randomized controlled trials. Int J Antimicrob Agents 62:106830. doi:10.1016/j.ijantimicag.2023.106830 37100354

[24] Zhang Y, Liu M, Zhang J, Wu J, Hong L, Zhu L, Long J. 2024. Large-scale comparative analysis reveals phylogenomic preference of blaNDM-1 and blaKPC-2 transmission among Klebsiella pneumoniae. Int J Antimicrob Agents 64:107225. doi:10.1016/j.ijantimicag.2024.107225 38810941

[25] Gu Y, Wang X, Zhang W, Weng R, Shi Q, Hou X, Wang H, Deng M, Mou J, Jiang Y. 2025. Dissemination of bla(NDM)-harboring plasmids in carbapenem-resistant and hypervirulent Klebsiella pneumoniae. Microbiol Spectr 13:e0196824. doi:10.1128/spectrum.01968-24 39936929 PMC11878072

[26] Li X, Li C, Zhou L, Wang Q, Yao J, Zhang X, Yu Y, Li R, Zhou H, Tu Y. 2024. Global phylogeography and genomic characterization of blaKPC and blaNDM-positive clinical Klebsiella aerogenes isolates from China, 2016-2022. Science of The Total Environment 923:171560. doi:10.1016/j.scitotenv.2024.171560 38458455

[27] Smillie C, Garcillán-Barcia MP, Francia MV, Rocha EPC, de la Cruz F. 2010. Mobility of plasmids. Microbiol Mol Biol Rev 74:434–452. doi:10.1128/MMBR.00020-10 20805406 PMC2937521

[28] Gong G, Chen Q, Luo J, Wang Y, Li X, Zhang F, Zhang Z, Cheng J, Xiong X, Hu R, Zhou Y. 2023. Characteristics of a ceftadine/avibatam resistance KPC-33-producing Klebsiella pneumoniae strain with capsular serotype K19 belonging to ST15. J Glob Antimicrob Resist 35:159–162. doi:10.1016/j.jgar.2023.09.013 37751846

[29] Shi Q, Yin D, Han R, Guo Y, Zheng Y, Wu S, Yang Y, Li S, Zhang R, Hu F. 2020. Emergence and recovery of ceftazidime-avibactam resistance in blaKPC-33-harboring Klebsiella pneumoniae sequence type 11 isolates in China. Clin Infect Dis 71:S436–S439. doi:10.1093/cid/ciaa1521 33367577

[30] Gao H, Liu Y, Wang R, Wang Q, Jin L, Wang H. 2020. The transferability and evolution of NDM-1 and KPC-2 co-producing Klebsiella pneumoniae from clinical settings. EBioMedicine 51:102599. doi:10.1016/j.ebiom.2019.102599 31911273 PMC6948161

[31] Huang J, Yi M, Yuan Y, Xia P, Yang B, Liao J, Dang Z, Luo S, Xia Y. 2022. Emergence of a fatal ST11-KL64 tigecycline-resistant hypervirulent Klebsiella pneumoniae Clone Cocarrying bla(NDM) and bla(KPC) in plasmids. Microbiol Spectr 10:e0253922. doi:10.1128/spectrum.02539-22 36205391 PMC9769963

[32] Cameranesi MM, Morán-Barrio J, Limansky AS, Repizo GD, Viale AM. 2018. Site-specific recombination at XerC/D sites mediates the formation and resolution of plasmid co-integrates carrying a blaOXA-58- and TnaphA6-resistance module in Acinetobacter baumannii. Front Microbiol 9:66. doi:10.3389/fmicb.2018.00066 29434581 PMC5790767

[33] Cheng Y, Li Y, Yu R, Ma M, Yang M, Si H. 2022. Identification of novel tet(X3) variants resistant to tigecycline in Acinetobacter species. Microbiol Spectr 10:e0133322. doi:10.1128/spectrum.01333-22 36409072 PMC9784759

[34] Sun J, Yang RS, Zhang Q, Feng Y, Fang LX, Xia J, Li L, Lv XY, Duan JH, Liao XP, Liu YH. 2016. Co-transfer of bla_NDM-5_ and mcr-1 by an IncX3-X4 hybrid plasmid in Escherichia coli. Nat Microbiol 1:16176. doi:10.1038/nmicrobiol.2016.176 27668643

[35] Yang X, Dong N, Chan EW-C, Zhang R, Chen S. 2021. Carbapenem resistance-encoding and virulence-encoding conjugative plasmids in Klebsiella pneumoniae. Trends Microbiol 29:65–83. doi:10.1016/j.tim.2020.04.012 32448764

[36] Song JM, Long HB, Ye M, Yang BR, Wu GJ, He HC, Wang JL, Li HW, Li XG, Deng DY, Li B, Yuan WL. 2024a. Genomic characterization of a blaKPC-2–producing IncM2 plasmid harboring transposon ΔTn6296 in Klebsiella michiganensis. Front Cell Infect Microbiol 14:1492700. doi:10.3389/fcimb.2024.1492700 39600872 PMC11588702

[37] Yuan M, Guan H, Sha D, Cao W, Song X, Che J, Kan B, Li J. 2021. Characterization of blaKPC-2-Carrying Plasmid pR31-KPC from a Pseudomonas aeruginosa strain isolated in China. Antibiotics (Basel) 10:1234. doi:10.3390/antibiotics10101234 34680814 PMC8532800

[38] Tang C, Shen S, Yang W, Shi Q, Ding L, Han R, Hu F. 2024. Dynamic evolution of ceftazidime-avibactam resistance from a single patient through the IncX3_NDM-5 plasmid transfer and bla_KPC_ mutation. Int J Antimicrob Agents 64:107228. doi:10.1016/j.ijantimicag.2024.107228 38823494

[39] Pu D, Zhao J, Lu B, Zhang Y, Wu Y, Li Z, Zhuo X, Cao B. 2023. Within-host resistance evolution of a fatal ST11 hypervirulent carbapenem-resistant Klebsiella pneumoniae. Int J Antimicrob Agents 61:106747. doi:10.1016/j.ijantimicag.2023.106747 36758779

[40] Song S, Yang S, Zheng R, Yin D, Cao Y, Wang Y, Qiao L, Bai R, Wang S, Yin W, Dong Y, Bai L, Yang H, Shen J, Wu C, Hu F, Wang Y. 2024b. Adaptive evolution of carbapenem-resistant hypervirulent Klebsiella pneumoniae in the urinary tract of a single patient. Proc Natl Acad Sci USA 121:e2400446121. doi:10.1073/pnas.2400446121 39150777 PMC11363291

