# Supplementary figures and images for: Within-host evolution drives the emergence of ceftazidime-avibactam resistance mediated by IncN plasmid-encoded blaNDM-1 and blaKPC-33 in ST11-KL64 hypervirulent Klebsiella pneumoniae
Source: Microbiol Spectr. 2026 Feb 19;14(4):e02367-25. doi: 10.1128/spectrum.02367-25 (PMC13055322; doi:10.1128/spectrum.02367-25)

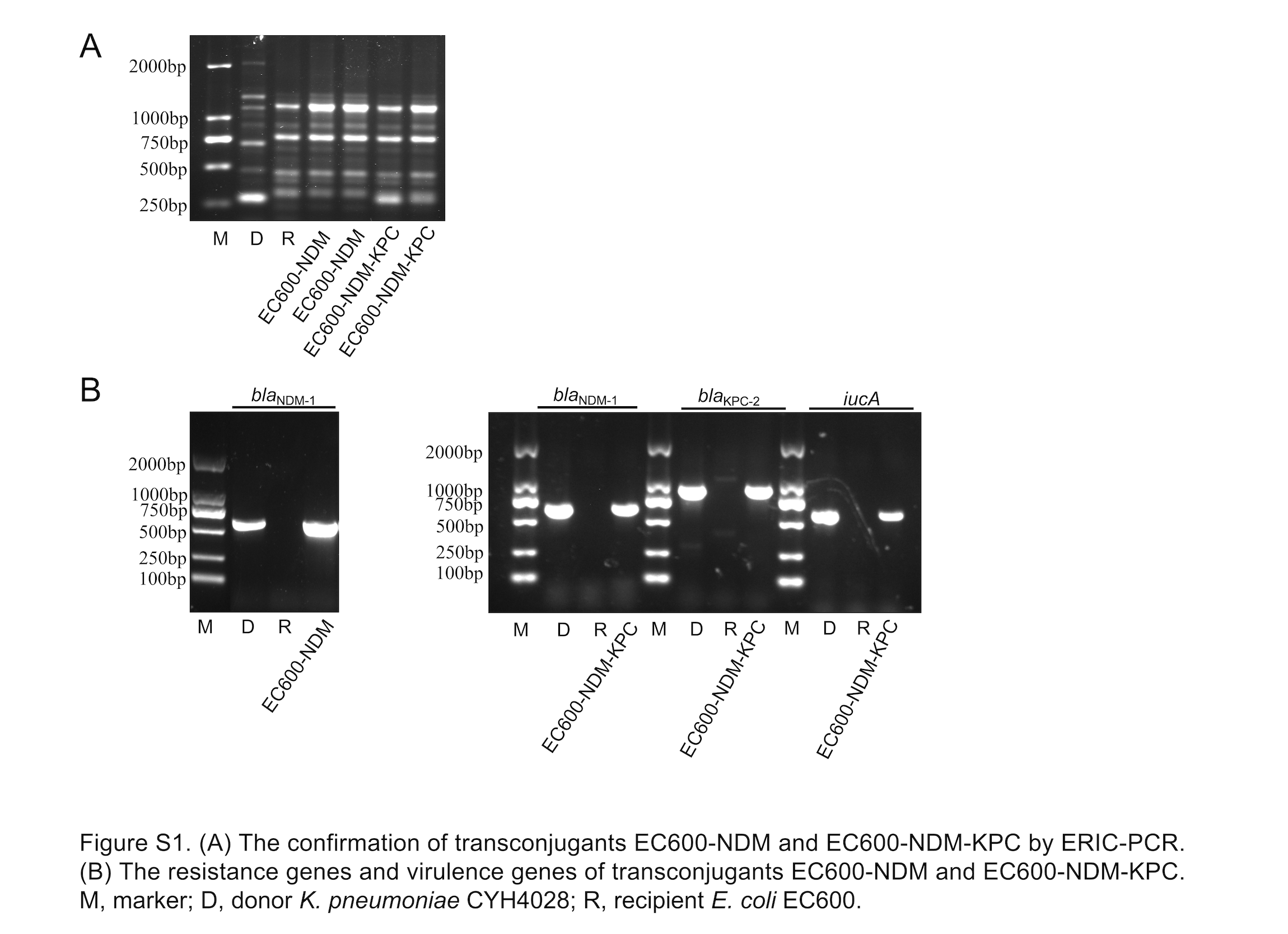

Supplement: Figure S1 — The confirmation of transconjugants EC600-NDM and EC600-NDM-KPC. [file spectrum.02367-25-s0001.tif]

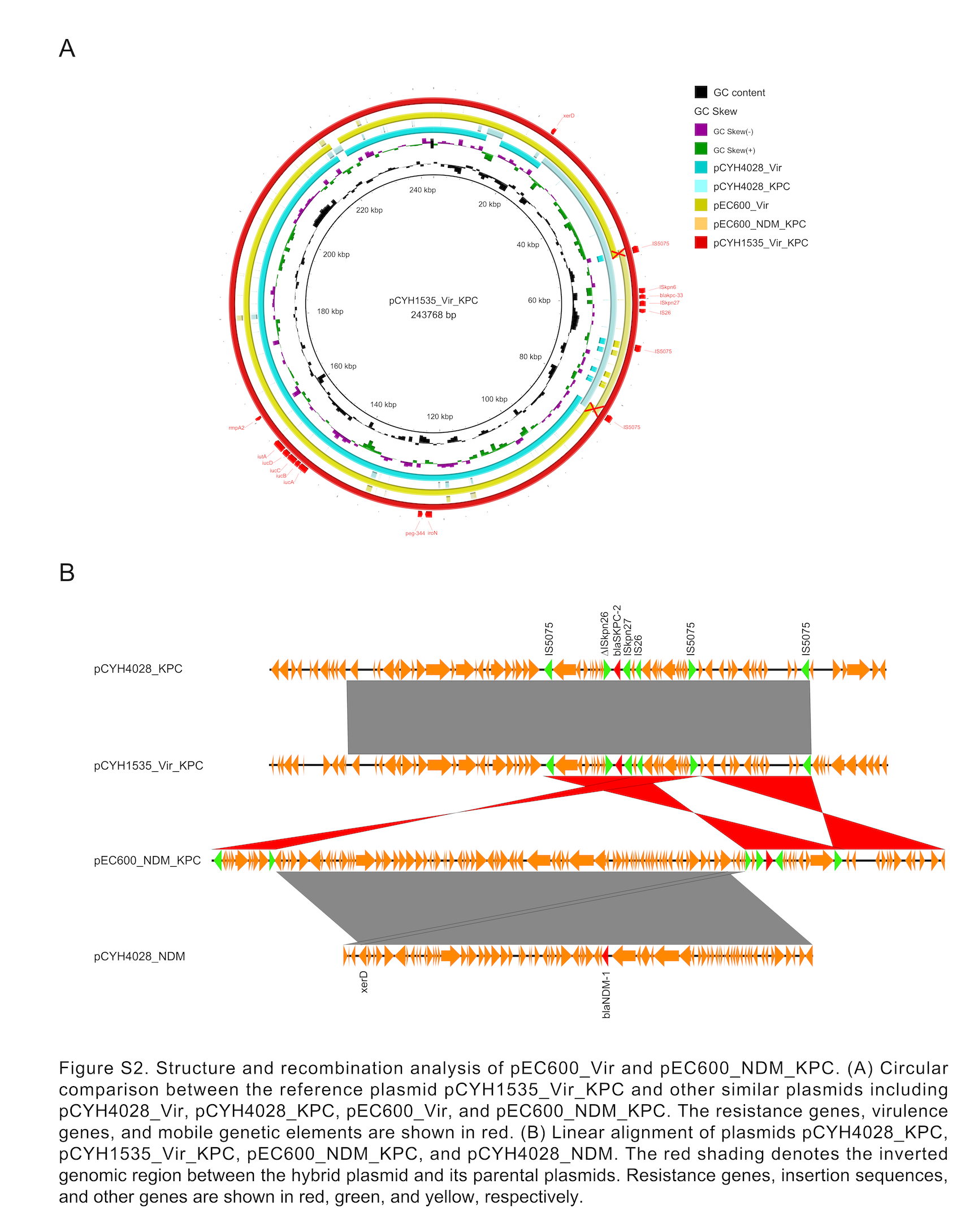

Supplement: Figure S2 — Structure and recombination analysis of pEC600_Vir and pEC600_NDM_KPC. [file spectrum.02367-25-s0002.tif]
