# Supplementary material for: Within-host evolution drives the emergence of ceftazidime-avibactam resistance mediated by IncN plasmid-encoded blaNDM-1 and blaKPC-33 in ST11-KL64 hypervirulent Klebsiella pneumoniae
Source: Microbiol Spectr. 2026 Feb 19;14(4):e02367-25. doi: 10.1128/spectrum.02367-25 (PMC13055322; doi:10.1128/spectrum.02367-25)
Supplement: Table S1 — Primers used in this study. [file spectrum.02367-25-s0003.docx]

| Primers | Primer sequences |
| --- | --- |
| Virulence genes | |
| *rmpA*-F | 5’- GAGTAGTTAATAAATCAATA-3’ |
| *rmpA*-R | 5’- CAGTAGGCATTGCAGCA-3’ |
| *rmpA2*-F | 5’- GTGCAATAAGGATGTTACATTA-3’ |
| *rmpA2*–R | 5’- GGATGCCCTCCTCCTG-3’ |
| *iucA*–F | 5’- GCTTATTTCTCCCCAACCC-3’ |
| *iucA*–R | 5’- TCAGCCCTTTAGCGACAAG-3’ |
| *iroB*-F | 5’- ATCTCATCATCTACCCTCCGCTC-3’ |
| *iroB*-R | 5’- GGTTCGCCGTCGTTTTCAA-3’ |
| *peg-344*-F | 5’-CTTGAAACTATCCCTCCAGTC-3’ |
| *peg-344*-R | 5’- CCAGCGAAAGAATAACCCC-3’ |
| Antibiotic resistance genes | |
| *bla*_KPC_-F | 5’- ATGTCACTGTATCGCCGTCT-3’ |
| *bla*_KPC_-R | 5’- TTACTGCCCGTTGACGCC-3’ |
| *bla*_NDM_-F | 5’- ATGGCTCATCACGATCATGC -3’ |
| *bla*_NDM_-R | 5’- AAGTGTGCTGCCAGACATTC-3’ |
| *bla*_CTX-M-9_-F | 5’- GGGCTGAGATGGTGACAAAGAG-3’ |
| *bla*_CTX-M-9_-R | 5’- CGTGCGAGTTCGATTTATTCAAC-3’ |
| *bla*_TEM_-F | 5’- CATTTCCGTGTCGCCCTTATTC-3’ |
| *bla*_TEM_-R | 5’- CGTTCATCCATAGTTGCCTGAC-3’ |
| *bla*_SHV_-F | 5’- AGCCGCTTGAGCAAATTAAAC-3’ |
| *bla*_SHV_-R | 5’- ATCCCGCAGATAAATCACCAC-3’ |

**Table S1. Primers used in this study.**
